# Treatment Trends and Epidemiologic Changes in Acetabular Fracture Management over the Course of 10 Years: An Analysis Based on 2853 Patients as Treated by the German Pelvic Multicenter Study Group

**DOI:** 10.3390/jcm13164601

**Published:** 2024-08-06

**Authors:** Silvan Wittenberg, Daniel Rau, Melissa Paraskevaidis, Vera Jaecker, Ulrich Stöckle, Sven Märdian

**Affiliations:** 1 Center for Musculoskeletal Surgery, Charité–Universitätsmedizin Berlin, Corporate Member of Freie Universität Berlin and Humboldt-Universität zu Berlin, 10117 Berlin, Germany; 2 Department of Trauma, Hand and Reconstructive Surgery, Rostock University Medical Center, 18057 Rostock, Germany

**Keywords:** acetabular fractures, German Pelvic Multicenter Study Group, epidemiology, treatment, trends, geriatric, trauma

## Abstract

**Background/Objectives:** Acetabular fractures, traditionally linked to high-impact trauma in younger adults, are increasingly observed in the elderly due to falls and poor bone quality. This demographic shift necessitates updated treatment approaches. This study analyzes demographic trends and treatment evolution over a decade using the German pelvic fracture registry. **Methods:** Data on acetabular fractures were analyzed from the German pelvic fracture registry of the German Trauma Society. Parameters included classification, demographics, treatment methods, and surgical details. Trends were assessed by grouping patients based on treatment intervals and age, comparing treatment methods, surgical approaches, and reduction quality across these groups, considering fracture types and treatment volume. **Results:** The study included 2853 unilateral acetabular fractures with a mean patient age of 61.5 years, showing an increasing age trend. A shift from simple to complex fractures involving the anterior acetabular column was observed. Operative treatment was performed in 62.5% of cases, more common in non-geriatric patients and those with posterior column involvement. The use of anterior intrapelvic approaches increased over time, replacing the Ilioinguinal extrapelvic approach. Anatomical reduction was achieved in 47.4% of cases, with 31.7% having imperfect reductions and 20.9% poor reductions. High-volume centers had significantly better reduction outcomes, particularly for simple fractures. Geriatric patients exhibited worse reduction quality compared to younger patients. In-hospital mortality was stable at 3.3%. **Conclusions:** The study highlights a demographic shift towards an older patient population, leading to more complex fracture patterns. Despite advancements in surgical techniques and new implant technologies, these demographic changes have resulted in lower reduction quality for complex fractures. Emphasis is placed on careful patient selection for reconstructive surgery or endoprosthetic replacement to ensure high-quality outcomes.

## 1. Introduction

Acetabular fractures exhibit a low incidence, ranging from 3 to 11 per 100,000 person-years and predominantly affect younger individuals following high-energy traumatic events [1,2,3]. However, an escalating trend is observed in the elderly population due to pre-existing osteoporosis and falls from a lower height [4]. In clinical practice, there is a continuous rise in the total number of such fractures within the geriatric demographic, often presenting with distinct fracture configurations.

High-energy trauma in younger patients is frequently associated with posterior acetabular column fractures or complex two-column disruptions, whereas low-energy falls in geriatric patients more commonly result in anterior column posterior hemitransverse fractures, often accompanied by concomitant acetabular dome impaction [5]. Recognizing this demographic shift, researchers have delved into this phenomenon, acknowledging its substantial implications for treatment strategies and modalities. In 2010, Ochs et al. conducted a comprehensive analysis of a large cohort of acetabular fracture cases. Their investigation yielded significant insights into the treatment, epidemiology, clinical presentation, and radiographic characteristics of these fractures. The study was based on data collected by the German Pelvic Multicenter Study Group of the German Trauma Society (DGU) over a 15-year period (1991–2006) [6]. 

The present study leverages data obtained from the same registry, encompasses the period from 2008 to 2017. It aims to provide a modernized comprehension of the contemporary landscape of acetabular fracture management. Additionally, the study aims to discern any trends or alterations that may have transpired over the elapsed decade. By capitalizing on the same data registry as the prior study, a direct comparison of the former findings is facilitated, thereby fostering a more holistic understanding.

## 2. Materials and Methods

This study is a retrospective cohort study and is based on data from the German pelvic fracture registry of the German Trauma Society (Deutsche Gesellschaft für Unfallchirurgie). The database is hosted domestically by the AUC (AUC—Akademie der Unfallchirurgie GmbH, Munich, Germany; https://www.auc-online.de, accessed on 24 November 2020) in strict accordance with the General Data Protection Regulation (GDPR) and local ethics committee approval in each federal state. Each of the 43 participating institutions, including all major university hospitals, entered data only pseudonymized and with informed patient consent.

**Figure 1:**
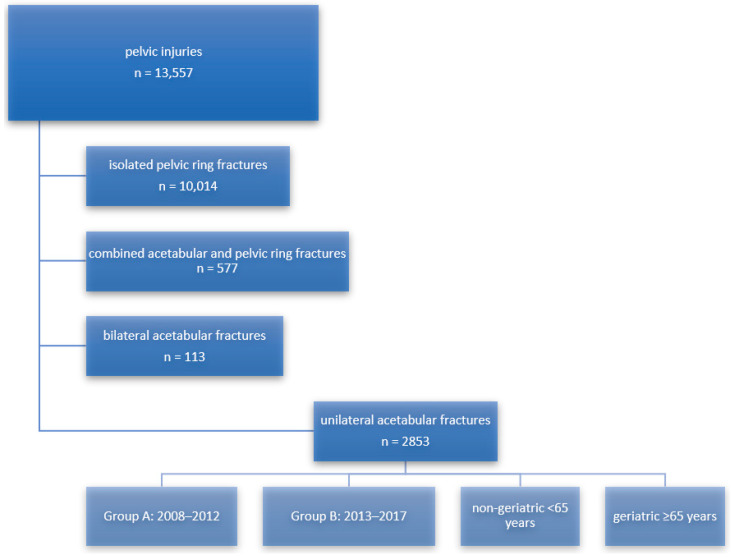
Depiction of the study sample consisting of 2853 unilateral acetabular fractures from a total of 13,557 pelvic injuries recorded between 2008 and 2017, according to the pelvic fracture registry of the German Trauma Society.

Inclusion criteria were unilateral acetabular fractures regardless of age, gender, trauma mechanism and type of treatment that occurred between January 2008 and December 2017 and were reported by the participating institutions. Exclusion criteria entailed bilateral acetabular fractures or acetabular fractures with combined pelvic ring fractures. 13,557 pelvis injuries were reported in the given period, of which 3543 were acetabular fractures. 2853 cases were unilateral and therefore included. Combined fractures of the acetabulum and pelvic ring (*n* = 577) and bilateral acetabular fractures (*n* = 113) were excluded from further analysis. An overview of the study sample is given in Figure 1.

All fractures had been classified using the AO/OTA fracture classification based on Letournel et al.’s fracture classification [7,8]. Baseline characteristics (age, sex), injury severity based on the Injury Severity Score [9], and treatment (operative versus non-operative) were recorded. Furthermore, surgical details (operation time, approaches, implants, time to operation, complications, mortality) and reduction quality were assessed in postoperative CT-scans according to Matta’s criteria (anatomical [0–1 mm displacement], imperfect [2–3 mm displacement], or poor [>3 mm displacement]) by the participating institutions [10]. To account for changes over the course of the study period the cohort was divided into two groups. Group A contained all patients that were treated from 2008–2012, Group B all patients from 2013–2017. Furthermore, to analyze for changes regarding patient age another two groups were created: a geriatric group consisting of patients ≥ 65 years of age and a non-geriatric group < 65 years of age. To compare treatment modality, surgical approaches and reduction quality groups were formed accordingly and additionally stratified by fracture types (simple versus associated) as well as case load by institution.

### Statistical Analysis

Normal distribution was tested using the Kolmogorov-Smirnov test. In the case of con-tinuous variables, the mean and standard deviation were used. In all other cases, the median and range are given. For statistical testing, the Kruskal-Wallis, Mann-Whitney-U, and Chi-square test were used to compare differences between samples according to data scales and number of groups. All statistical analyses were performed using SPSS 28.0 software (IBM Inc., Armonk, New York, USA). *p* < 0.05 was considered statistically significant.

## 3. Results

### 3.1. Demographics

**Figure 2:**
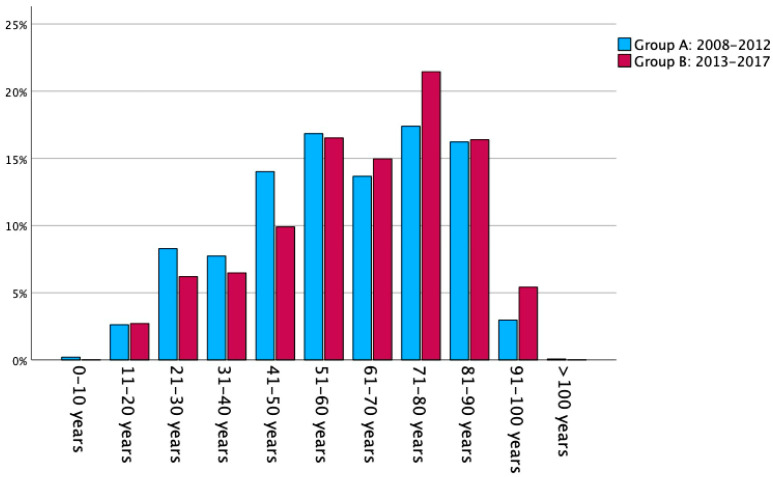
Age distribution of patients in percentage brackets for the study cohorts from 2008–2012 (Group A: *n* = 1449) and 2013–2017 (Group B: *n* = 1404); total *n* = 2853.

There were 2853 unilateral acetabular fractures (Group A: 1449 patients; Group B: 1404 patients), with 815 (28.6%) female and 2038 (71.4%) male patients, respectively. The mean age was 61.5 ± 20.4 years with a mean ISS of 12.3 ± 7.1 points. 1394 (48.9%) patients were at least 65 years old and considered geriatric. The age ranged between 8 and 105 years, with a peak in the 70–80-year interval (Figure 2). Data revealed a significant increase in mean patient age (Group A: 60.0 ± 20.6 years versus Group B: 63.1 ± 20.1 years; *p* < 0.001) over the study period. In this context, males were significantly younger than females (58.0 ± 19.4 years versus 68.5 ± 20.8 years; *p* < 0.001). The gender distribution remained stable over the study period (*p* = 0.365). Basic study population characteristics, divided by injury severity and gender, are represented in Table 1.

### 3.2. Fracture Classification

Fracture classification revealed 1380 (48.1%) simple and 1472 (51.9%) associated acetabular fractures according to the Letournel classification. Within the simple fracture patterns, anterior pathologies were the leading entities, followed by dorsal pathologies and transverse fractures. The leading fracture morphologies in the associated group were the anterior column posterior hemitransverse and both columns injuries (Table 2). When comparing the two time intervals in this regard, data revealed a shift from primarily simple to associated fractures (*p* < 0.001). Specifically, anterior column posterior hemi-transverse and both columns fractures increased percentagewise while simple anterior column fractures decreased (Table 2).

**Figure 3:**
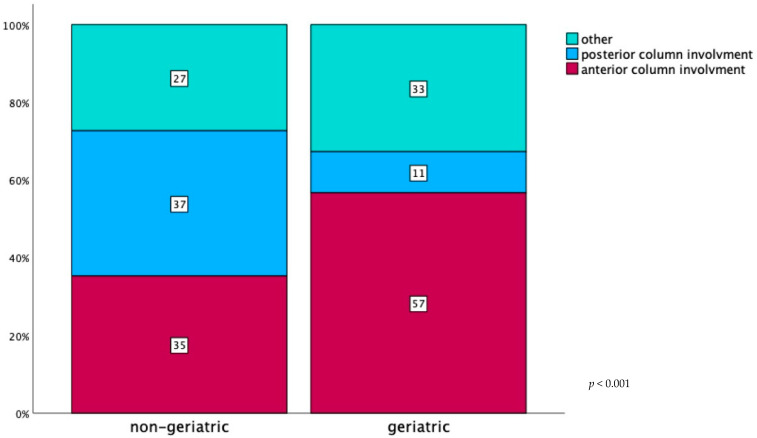
Percentage of acetabular fracture types involving the anterior (anterior wall, anterior column, anterior column + posterior hemitransverse) and posterior column (posterior wall, posterior column, posterior column + posterior wall, transverse fracture with posterior wall) divided by age: geriatric (age ≥ 65 years: *n* = 1394) and non-geriatric (age < 65 years: *n* = 1459).

Anterior fracture types (anterior wall, anterior column, anterior column posterior hemitransverse) occurred in 1305 (46%) cases overall. In comparison, in 693 (24%) patients, a posterior fracture type was observed (posterior column, posterior wall, posterior column posterior wall, transverse fracture plus posterior wall). When analysing this distribution by age, a high number of fractures only affecting the anterior acetabular column (*n* = 790 (57%)) compared to the posterior column (*n* = 148 (11%)) was seen in the geriatric group. In non-geriatric patients, the numbers of anterior versus posterior column involvement were almost equal (*n* = 515 (35%) and *n* = 545 (37%) respectively; *p* < 0.001; Table 3, Figure 3). Associated fractures were more common in the geriatric group (55.9% versus 47.6%, *p* < 0.001). 

### 3.3. Operative and Nonoperative Treatment

**Figure 4:**
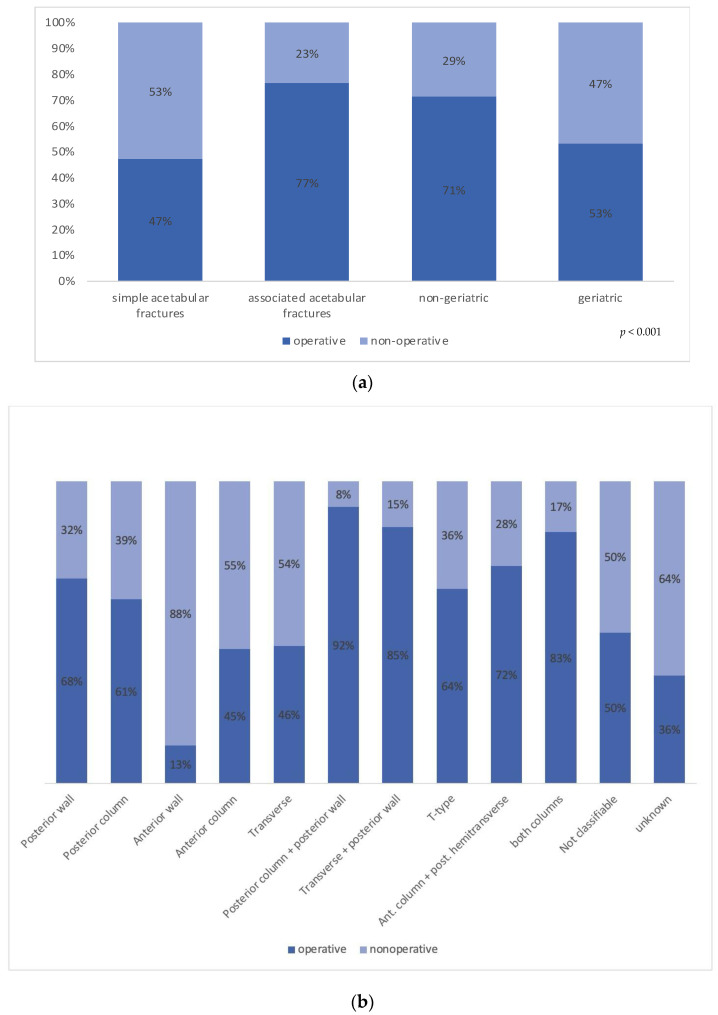
(**a**) Treatment distribution of acetabular fractures by percentage, categorized by fracture complexity (simple: *n* = 1380; associated: *n* = 1473) and patient age: geriatric (age ≥ 65 years: *n* = 1394) and non-geriatric (age < 65 years: *n* = 1459); (**b**) Treatment distribution of all acetabular fracture types (*n* = 2853) according to the Letournel classification, presented in percentage.

1783 (62.5%) patients, averaging 178.3 cases per year, were treated surgically. The mean age was 58.5 ± 19.5 years, with a mean ISS of 12.2 ± 6.6. In contrast, 1,070 (37.5%) patients with a significantly higher mean age (66.5 ± 20.7 (*p* < 0.01)) were treated nonoperatively with a mean ISS of 12.5 ± 7.8 points. Distributed by age, geriatric patients were less often treated operatively than non-geriatric patients (724 (53.2%) versus 1,041 (71.4%); *p* < 0.001, Figure 4a). On the other hand, associated fracture types were treated surgically more often than simple fracture types (1129 cases (76.6%) versus 654 cases (47.4%), *p* < 0.001, Figure 4a). Over the whole study period, the treatment distribution remained stable (*p* = 0.904).

Subgroup analysis revealed that posterior column and/or posterior wall fractures had the highest surgery rate (92%). The complete overview of the subgroup analysis is given in Figure 4b.

### 3.4. Surgical Approaches

The Ilioinguinal approach emerged as the most widely utilized anterior approach to the acetabulum (35%). Intrapelvic approaches, i.e., the (modified) Stoppa and Pararectus approach, were used in 12% and 6%, respectively. The Smith-Peterson approach was utilized in the minority of cases (1%). Closed reduction and percutaneous techniques were used in 7% of the cases. 32% of the patients required a posterior approach, using the Kocher-Langenbeck approach. Surgical approaches were utilized individually, in com-bination, or staged.

**Figure 5:**
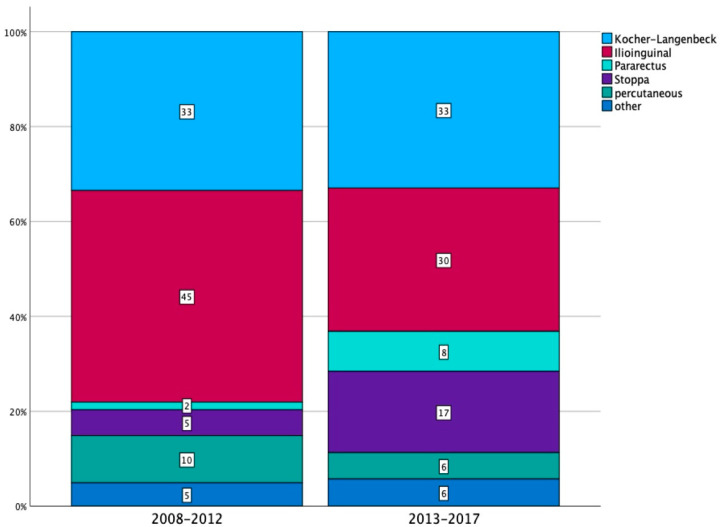
Distribution of surgical approaches to the acetabulum (*n* = 2034 in 1783 operatively treated patients), comparing the time periods 2008–2012 (Group A: *n* = 1019) and 2013–2017 (Group B: *n* = 1015).

Over the study period, a notable rise in the use of anterior intrapelvic approaches (Stoppa and Pararectus) was evident, which came at the cost of the Ilioinguinal extrapelvic approach (*p* < 0.001, see Figure 5). While the Kocher-Langenbeck approach remained steady throughout the study period, minimally invasive percutaneous treatments saw a decline.

### 3.5. Implants

Fracture fixation was most often performed by pelvic reconstruction plates with free 3.5mm cortex screws in *n* = 1399 (80.8%) cases. Locking plates were used in *n* = 31 (1.8%) of the cases only. Percutaneous screws were applied in *n* = 157 (9.1%). A total of *n* = 78 (4.5%) patients required a primary total hip arthroplasty, of which *n* = 21 (1.2%) needed additional osteosynthetic reconstruction. Other implants were used in *n* = 12 (0.7%) patients. Furthermore, documentation was insufficient in *n* = 54 (3.1%) cases. When comparing the two intervals, there was a significant decline in minimally invasive percutaneous screw fracture fixation (2008–2012 (Group A): 101 versus 2013–2017 (Group B): 54; *p* = 0.001). As expected, patients treated with a primary THA averaged 75 ± 13 years of age, whereas patients undergoing joint reconstruction averaged 57 ± 19.4 years (*p* = 0.001). 

### 3.6. Time to Operation and Operation Time

The mean time to surgery was 4.9 ± 3.7 days, and the mean OR (operating room) time revealed 178 ± 86 minutes. Most patients (*n* = 1499, 84.1%) could be operated on within the first seven days following injury, whereas *n* = 239 (13.4%) were treated within 8–14 days, and in *n* = 45 (2.5%) cases, surgery was delayed beyond 14 days. There was no significant change of either time to surgery or OR time in the two intervals (*p* = 0.270 and *p* = 0.209 respectively).

Regarding the fracture types, the analysis revealed that simple fractures were stabilized significantly earlier than associated fractures (4.7 ± 3.7 versus 5.1 ± 3.7 days; *p* = 0.006). Furthermore, OR time was significantly longer for associated fractures compared to simple fractures (195 ± 92 versus 150 ± 67 minutes; *p* < 0.001).

### 3.7. Fracture Reduction

**Figure 6:**
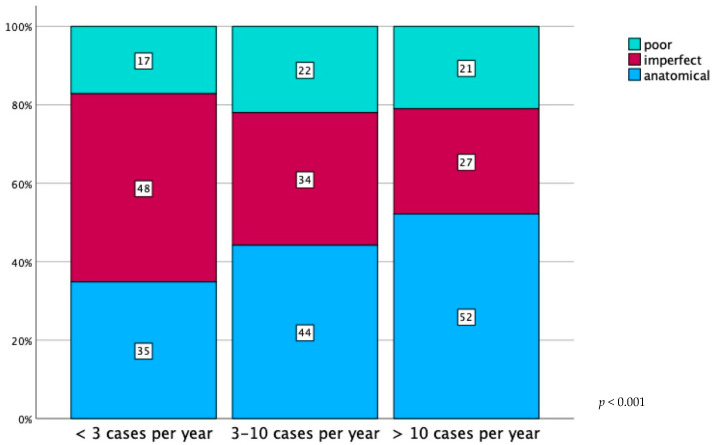
Percentage distribution of postoperative acetabular fracture reduction grades, as defined by Matta et al., stratified by annual surgery counts reported by the participating institutions (<3 cases per year: *n* = 221; 3–10 cases per year: *n* = 582; >10 cases per year: *n* = 980).

Postoperative fracture reduction assessment was available in 1783 cases. In 845 (47.4%) patients, an anatomical reduction was achieved, and 566 (31.7%) and 372 (20.9%) cases showed an imperfect and poor reduction, respectively. When investigating for operative case count by clinic per year in regard to fracture reduction quality our analysis revealed significantly better outcomes in high-volume centers (*p* < 0.001; Figure 6). There was no evidence for changes in fracture reduction quality over the study period (*p* = 0.553).

**Figure 7:**
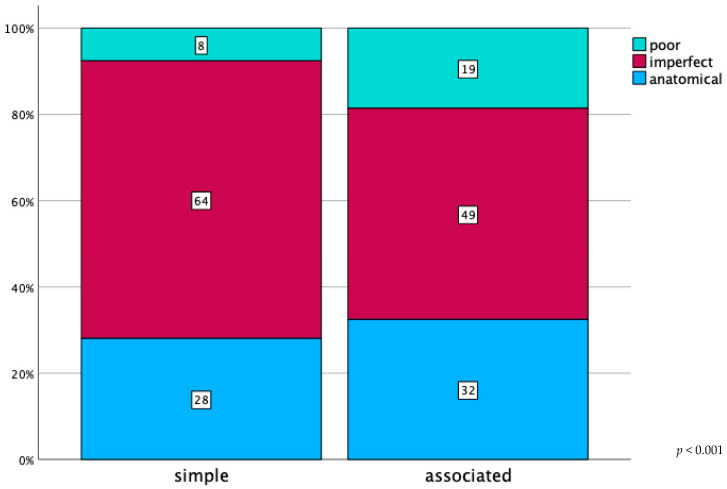
Postoperative acetabular fracture reduction grading in percentage, as defined by Matta et al., categorized by fracture complexity (simple: *n* = 654; associated: *n* = 1129, as defined by Letournel).

**Figure 8:**
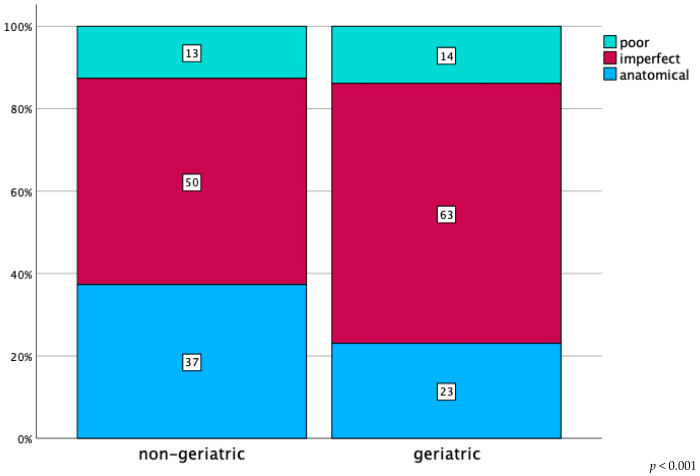
Postoperative acetabular fracture reduction grading in percentage, as defined by Matta et al., categorized by patient age (geriatric: *n* = 742; non-geriatric: *n* = 1041).

Simple fracture types were significantly better reduced compared to the associated fractures (*p* < 0.001; Figure 7) When analysing the reduction quality in terms of patient age, data showed that geriatric patients had significantly worse reduction quality than the non-geriatric group (*p* < 0.001; Figure 8). There was no statistically significant difference in reduction quality among the principal anterior approaches (Ilioinguinal versus Pararectus versus Stoppa; *p* = 0.563). All data is presented in Table 4.

### 3.8. Complications and Mortality

The in-hospital overall complication rate in the operative group was high at 22.6% (404/1783). Compared to this, the complication rate in the non-operative group was only 9.1% (101/1070). In-hospital mortality was 3.3% (*n* = 95), of which 41 patients were treated surgically and 54 patients non-operatively. A detailed overview of the documented complications is provided in Table 5. There was no notable change in rates of complication or mortality over the course of the study period (*p* = 0.495 and *p* = 0.081 respectively).

## 4. Discussion

This retrospective study was conducted on data from 2853 patients diagnosed with unilateral acetabular fractures. The data were obtained from 43 participating hospitals within the German Pelvic Multicenter Study Group, a collaborative effort of the German Trauma Society (DGU) and evaluated from January 2008 to December 2017. 

Our results reflect the ongoing demographic change with a constantly increasing patient age, which is even more pronounced when comparing our data to Ochs et al., who reported a mean patient age of only 47.3 ± 20.1 years in 2010 based on the same registry [6]. This shift in patient age and the increasing number of patients suffering from these fractures have already been described by other authors and identified as a severe public health issue in an aging population [2,6,11]. Some authors consider pelvic injuries as a whole as the fastest-growing patient cohort in orthopedic trauma care [2,12,13]. In our study, the majority of patients with acetabular fractures were men, accounting for 71.4% of the study population, with a steady gender distribution over the study period. Other authors have also observed a male predominance in acetabular fractures, ranging from 54% to 77% [2,5,9]. At the same time, male patients are, on average, nearly a decade younger than their female counterparts. This stands in sharp contrast to femoral neck fractures, where the gender distribution is flipped. The cause behind this distinct gender discrepancy remains unclear to date. The change in age is accompanied by a shift in fracture patterns from simple to more complex associated fracture types [5]. Our data confirm that geriatric patients most commonly sustained fractures involving the anterior acetabular column, especially combined with a posterior hemitransverse fracture or both columns fractures. At the same time, fractures with involvement of the posterior acetabular column declined drastically with increasing age. These results compare well to data published elsewhere, and the trend is expected to continue [11].

Operative treatment remained the primary choice for displaced and unstable acetabular fractures in suitable and mostly younger patients, with 62.7% of all cases were treated surgically. Geriatric patients were predominantly managed non-operatively. Notably, the surgery rate was higher than in other European countries (e.g., France, Belgium, and Sweden). Based on data from nationwide registries in those countries, operative treatment rates range from only 12% to 15% [2,14,15]. Remarkably, those registries report low surgery rates even for non-geriatric patients. 

Our non-operative treatment rate is somewhat higher than the 32.2% reported by Ochs et al., a difference that could be attributed to the higher mean age of patients in our study. However, despite an increase in average patient age, the proportion of operative versus non-operative treatments remained stable during the study period [6].

Our analysis of fracture types revealed that predominantly associated acetabular fractures underwent surgery compared to simple fractures, with the goal of anatomical fracture reduction. Previous research has already shown that the quality of reduction and restoration of the acetabular dome and posterior wall significantly reduces the long-term risk of developing osteoarthritis, with rates as high as 80% [10,16,17,18]. Anatomical reduction was achieved in 47.4%, imperfect reduction in 31.7%, and poor reduction in 20.9% of the cases - a distribution that has remained constant in both study intervals. Comparing these data to Ochs et al. (anatomical 74.8%, imperfect 11.9%, poor 6.2%, not documented 7.1%) demonstrates that reduction quality tremendously decreased in the registry over the last decades [6]. In contrast, other authors in high volume centers reported anatomical reduction rates between 67% and 73.9% in the past [10,16,17]. However, like Ochs et al., these data also reflect patient cohorts with a significantly lower mean patient age ranging from 37 to 46 years which has substantial influence on fracture complexity as shown above. In addition to age and fracture complexity, there was a significant relationship between the number of surgeries performed by a specific institution and the graded reduction quality. We therefore believe that referral to a trauma center with high experience in acetabular surgery for definitive treatment is warranted.

The evolution towards anterior pathologies necessitates appropriate surgical access. Especially dome impaction and medial femoral head dislocations in anterior column posterior hemitransverse fractures may lead to non-anatomical fracture reductions due to limited options to stabilize these anatomical structures sufficiently. Therefore, many attempts to improve the reduction quality by using intrapelvic approaches and specialized implants designed to support the quadrilateral surface have been made. For instance, the intrapelvic Stoppa and Pararectus approaches provide excellent visualization of the anterior column and quadrilateral surface when compared to the established Ilioinguinal approach [19,20,21,22]. In our study, the change in fracture types and complexity merges well with the surgeons’ preference for less invasive intrapelvic surgical approaches. 

Open reduction and plate fixation were the leading procedures in treating acetabular fractures. Sometimes closed reduction and percutaneous or minimally invasive screw or plate osteosyntheses (MIPO) techniques can be utilized. However, associated fractures of the acetabulum are often ineligible for closed reduction due to anatomical circumstances and dome impaction. This might explain the relatively low and even declining rate of percutaneous techniques. However, new attempts utilizing laparoscopy have recently been made to establish minimally-invasive operating procedures in acetabular surgery but have not yet been widely used in clinical practice [23]. In addition to osteosynthesis, there are situations where a primary joint replacement might be a suitable alternative (e.g., accompanying femur head or neck fracture, marginal dome impaction, destruction of more than 40% of the load-bearing joint surface, severe osteoporosis) to restore mobility in elderly individuals immediately [24,25,26,27,28,29]. Although the mean age and typical complex fracture patterns of geriatric patients steadily increased, the number of primary joint replacements remained constant over time, even when compared to previous studies [6]. We believe this improvement can be attributed to the enhanced operating techniques mentioned earlier.

Both mortality and complication rates remained stable during the study interval and were on par with former results of Ochs et al [6]. Interestingly, patients in the non-operative group have been shown to have a two-fold higher risk of death during hospital stay. On the other hand, this patient group has significantly fewer complications than the operative group overall. These data reflect that patients’ general medical conditions still considerably impact the surgery indication.

This study’s strength is highlighted by several pivotal aspects. First, the large number of acetabular fracture cases contributes to robust statistical power and reliability of the results. The multicenter design enhances the generalizability of the findings by including all major hospitals and level I trauma centers, ensuring diverse patient demographics and clinical practices. The lengthy study period allows for comprehensive longitudinal analysis. The ability to compare findings directly with a predecessor study based on the same registry further strengthens the validity of the conclusions. Additionally, the high quality of data maintained throughout the study underscores the accuracy and reliability of the results.

However, this study also has some limitations. It is the nature of registry-based studies that they are retrospective with all their limitations. Furthermore, comparison of reduction quality with data of older studies is difficult, given the widespread availability of high-resolution CT scans for intraoperative and postoperative use nowadays, which can detect even minor persistent fracture dislocations, that may not have been visible in the past. Lastly, long-term outcome data of patients are missing, so the impact of surgical approach, quality of reduction, and complications on such outcomes cannot be evaluated.

## 5. Conclusions

This retrospective analysis examines epidemiological, clinical, and radiological data of 2853 patients with unilateral acetabular fractures over a ten-year period (2008–2017). The study reveals a shift in patient demographics and its subsequent impact on surgical treatment strategies. Notably, the demographic changes observed are consistent with a broader societal trend of rapid aging. This has led to a significant increase in a specific fracture pattern – the anterior column posterior hemitransverse fracture. This fracture type, in conjunction with factors such as patient age, comorbidities, and bone quality, presents challenges in achieving optimal reduction quality. However, the study also demonstrates an evolution in surgical approaches, with a notable increase in the utilization of intrapelvic approaches specifically tailored to address these emerging fracture patterns. Additionally, the introduction of new implant technologies has the potential to improve fracture fixation. Despite these advancements, our findings highlight new challenges associated with the changing demographics. The study demonstrates a significant decrease in reduction quality associated with the shift in fracture patterns and compromised bone stock. Moving forward, careful patient selection for either reconstructive surgery or endoprosthetic replacement will be crucial to ensure high-quality outcomes in the treatment of these fractures.

## Figures and Tables

**Table 1:** Mean patient age, mean patient age by gender, and mean ISS for the total study cohort, stratified by treatment interval, 2008–2012 (group A) and 2013–2017 (group B).

	Total Study Cohort (*n* = 2853)	Group A (*n* = 1449)	Group B (*n* = 1404)
Age (total)	61.5 ± 20.4	60.0 ± 20.6	63.1 ± 20.1
Age (female) Age (male)	68.5 ± 20.8 58.0 ± 19.4	68.0 ± 21.3 56.9 ± 19.5	70.6 ± 19.7 60.0 ± 19.4
ISS	12.3 ± 7.1	12.7 ± 7.4	12.0 ± 6.7

**Table 2:** Distribution of acetabular fractures classified by Letournel and Judet, categorized into simple and associated fractures. Data is presented both in total and stratified by treatment intervals 2008–2012 (Group A) and 2013–2017 (Group B).

Fracture Entity	Total	Group A	Group B
Simple fractures	1380 (48.1%)	678 (46.8%)	557 (39.7%)
Posterior wall Posterior column Anterior wall Anterior column Transverse	349 (12.2%)	184 (12.7%)	165 (11.8%)
	123 (4.3%)	63 (4.3%)	60 (4.3%)
	200 (7.0%)	104 (7.2%)	96 (6.8%)
	563 (19.7%)	327 (22.6%)	236 (16.8%)
	145 (5.1%)	89 (6.1%)	56 (4.0%)
Associated fractures	1473 (51.9%)	771 (53.2%)	847 (60.3%)
Posterior column + posterior wall Transverse fracture + posterior wall T-type Anterior column posterior hemitransverse	95 (3.3%)	43 (3.0%)	52 (3.7%)
	126 (4.4%)	67 (4.6%)	59 (4.2%)
	135 (4.7%)	66 (4.6%)	69 4.9%)
	542 (19.0%)	230 (15.9%)	312 (22.2%)
Both columns	519 (18.2%)	250 (17.3%)	269 (19.2%)
Unknown	14 (0.4%)	5 (0.3%)	2 (0.1%)
Not classifiable	42 (1.5%)	21 (1.4%)	28 (2.0%)
Total	2853 (100%)	1449 (50.7%)	1404 (49.3%)

**Table 3:** Acetabular fracture distribution as classified by Letournel and Judet for total study cohort and divided by age in non-geriatric (age < 65 years) and geriatric (age ≥ 65 years).

Fracture Entity	Total	Non-Geriatric	Geriatric
Simple fractures	1380 (48.1%)	765 (52.4%)	615 (44.1%)
Posterior wall Posterior column Anterior wall Anterior column Transverse	349 (12.2%)	289 (10.5%)	60 (4.3%)
	123 (4.3%)	73 (5.0%)	50 (3.6%)
	200 (7.0%)	81 (5.6%)	119 (8.5%)
	563 (19.7%)	242 (16.9%)	321 (23.0%)
	145 (5.1%)	80 (5.5%)	65 (4.6%)
Associated fractures	1473 (51.9%)	694 (47.6%)	779 (55.9%)
Posterior column + posterior wall Transverse fracture + posterior wall T-type Anterior column posterior hemitransverse	95 (3.3%)	77 (5.3%)	18 (1.3%)
	126 (4.4%)	106 (7.3%)	20 (1.4%)
	135 (4.7%)	58 (4.0%)	77 (5.5%)
	542 (19.0%)	192 (13.2%)	350 (25.0%)
Both columns	519 (18.2%)	242 (16.6%)	277 (19.8%)
Unknown	14 (0.4%)	1 (0.1%)	6 (0.4%)
Not classifiable	42 (1.5%)	18 (1.2%)	31 (2.2%)
Total	2853 (100%)	1459 (51.2%)	1394 (48.8%)

**Table 4:** Postoperative acetabular fracture reduction grading according to fracture complexity.

Reduction Grading	Number of Patients (%)
Simple fractures	
Anatomical (0–1 mm displacement) Imperfect (2–3 mm displacement) Poor (>3 mm displacement)	381 (58.3%)
	169 (25.8%)
	104 (15.9%)
Associated fractures	
Anatomical (0–1 mm displacement) Imperfect (2–3 mm displacement)	464 (41.1%)
	397 (35.2%)
Poor (>3 mm displacement)	268 (23.7%)

**Table 5:** Complications * and mortality of the study cohort.

	Operative (*n* = 1783)	Non-Operative (*n* = 1070)	Sig.
Total number of patients with at least one complication	404 (22.6%)	101 (9.4%)	<0.001
Total number of complications Deep vein thrombosis Embolism ARDS Nerve affection Multi-organ failure Infection superficial Bleeding/anaemia Wound healing disorder Infection deep Seroma Implant failure Implant loosening Secondary dislocation Haematoma Other	505 28 (1.6%) 20 (1.1%) 6 (0.3%) 74 (4.1%) 4 (0.2%) 19 (1.0%) 40 (2.2%) 7 (0.4%) 49 (2.7%) 10 (0.5%) 10 (0.5%) 10 (0.5%) 23 (1.2%) 36 (2.0%) 169 (9.5%)	133 5 (0.5%) 9 (0.8%) 10 (0.9%) 4 (0.4%) 14 (1.3%) 1 (0.1%) 8 (0.7%) 1 (0.1%) 6 (0.6%) 1 (0.1%) - - 2 (0.2%) 8 (0.7%) 64 (6.0%)	<0.001 0.008 0.471 0.038 <0.001 0.023 <0.001 0.003 0.144 <0.001 0.014 <0.001 <0.001 <0.002 0.008 <0.001
Mortality	41 (2.3%)	54 (5.0%)	<0.001

* Some patients suffered more than one complication.

## Data Availability

The data presented in this study are available on request from the corresponding author due to data privacy regulations.
